# The impact of multiple long-term conditions on mortality, progression to kidney failure and health-related quality of life among people with chronic kidney disease: a multicentre cohort study (NURTuRE-CKD)

**DOI:** 10.1080/07853890.2026.2692812

**Published:** 2026-06-23

**Authors:** Irene Boateng, Thomas Phillips, Scott Harris, Olalekan Lee Aiyegbusi, Paul Cockwell, Philip A. Kalra, Thomas McDonnell, Beth Lucas, Paul J. Roderick, David C. Wheeler, Maarten W. Taal, Simon D. S. Fraser

**Affiliations:** ^a^ School of Primary Care, Population Sciences and Medical Education, Faculty of Medicine, University of Southampton, Southampton General Hospital, Southampton, UK; ^b^ Centre for Patient-Reported Outcome Research (CPROR), University of Birmingham, Edgbaston, Birmingham, UK; ^c^ Department of Renal Medicine, Queen Elizabeth Hospital, University Hospitals of Birmingham, Edgbaston, Birmingham, UK; ^d^ Institute of Inflammation and Ageing, University of Birmingham, Edgbaston, Birmingham, UK; ^e^ Donal O’Donoghue Renal Research Centre, Salford Royal Hospital, Northern Care Alliance NHS Foundation Trust, Salford, UK; ^f^ Division of Cardiovascular Sciences, Faculty of Biology Medicine and Health, University of Manchester, Manchester, UK; ^g^ Centre for Kidney Research and Innovation, Academic Unit for Translational Medical Sciences, School of Medicine, University of Nottingham, Nottingham, UK; ^h^ Department of Renal Medicine, University College London, London, UK; ^i^ Department of Renal Medicine, Royal Derby Hospital, University Hospitals of Derby and Burton NHS Foundation Trust, Derby, UK

**Keywords:** Chronic kidney disease, multimorbidity, multiple long-term conditions, mortality, CKD progression, health-related quality of life

## Abstract

**Background:**

Multiple long-term conditions (MLTC) are common in people with chronic kidney disease (CKD). This study examined the impact of MLTC on mortality, CKD progression, and health-related quality of life (HRQoL) in a referred CKD population.

**Methods:**

Adults with non–kidney replacement therapy (KRT)-dependent CKD (stages G1 to G5) were recruited to the prospective NURTuRE-CKD cohort across 16 UK nephrology centres. MLTC was defined as ≥2 conditions (including CKD) and comorbidities categorised into 19 groups. Depression and anxiety were defined using the Hospital Anxiety and Depression Scale, and Cognitive impairment using the Six-item Cognitive Impairment Test. Outcomes were all-cause mortality, CKD progression (eGFR <15 mL/min/1.73m^2^ or KRT), and HRQoL (EQ-5D-5L, at two time points). Participants with eGFR < 15 at baseline were excluded from progression analyses.

**Results:**

All 2996 participants had comorbidities at baseline, and therefore MLTC. Mean age was 62.7 (SD ± 14.7) years, mean eGFR 37.3 mL/min/1.73m^2^ (SD ± 17.9), and 41% were female. Median baseline comorbidity count was 3 (IQR 2 to 5; range 1 to 19). The commonest baseline comorbidities were hypertension, pain, obesity, hyperuricaemia, diabetes, sarcopenia, and cardiovascular disease. Over a median 1.42 years between baseline and first follow up, the most frequent new comorbidities were pain (285 people (14%)), anxiety and depression (226 (11%)), cognitive impairment (115 (6%)), obesity (98 (5%)), and anaemia (82 (4%)). Increasing comorbidity count was associated with higher mortality (adjusted hazard ratios (vs. 1 comorbidity): 1.37 (2), 1.45 (3), 1.54 (4), and 1.80 (≥5)), with statistical significance at ≥4 comorbidities). Comorbidity number was not associated with CKD progression. Greater number and certain specific comorbidities were associated with worse HRQoL.

**Conclusions:**

Greater comorbidity burden was associated with mortality and worse HRQoL but not CKD progression in this cohort. CKD management should target prevention of additional conditions, prioritise holistic care and focus on MLTC as much as kidney protection.

## Abstract (plain language summary)

**What is already known on this topic:**

- Having multiple long-term conditions is common among people with CKD.
- People with CKD commonly have worse health-related quality of life (HRQoL) than similar populations without CKD.
- HRQoL is affected by many factors among people with CKD and often worsens over time.

**What this study adds:**

- Accruing new comorbidities over time is common among people with non-kidney replacement therapy (KRT)-dependent CKD, particularly pain and mental health conditions.
- Having a greater number of comorbidities was associated with mortality, but not progression to kidney failure among people with CKD in this particular population and period of follow up.
- A greater number and a wider range of specific comorbidities were associated with worse longitudinal HRQoL among people with CKD

**How this study might affect research, practice, or policy:**

- This research encourages holistic care of people with CKD with a focus on multiple long-term conditions being as important as kidney protection.
- Greater efforts are needed to address specific comorbidities, particularly pain, mental health conditions (anxiety and depression), cognitive impairment, obesity, sarcopenia, anaemia, and cardiovascular disease.
- This research supports preventative interventions that reduce the risk of developing new long-term conditions among people living with CKD.

## Introduction

Multiple long-term conditions (MLTC or ‘multimorbidity’, usually defined as the presence of two or more long-term conditions) are common among people with chronic kidney disease (CKD) [1,2]. Co-existence of CKD and comorbidities complicates management and has been associated with poor health-related quality of life (HRQoL), increased healthcare utilisation and cost, length of in-hospital stay and mortality [3–12]. Research to date on the relationship between MLTC and adverse clinical outcomes has focused largely on mortality among people with advanced CKD. A recent systematic review of 26 cohort studies included only six among people with non-kidney replacement therapy (KRT) -dependent CKD stage 3 to 5, and only one of those investigated CKD progression as an outcome [13,14].

Previous studies of MLTC and HRQoL among people with CKD have reported high comorbidity prevalence and documented the most prevalent conditions but have often been limited by their cross-sectional nature and/or considering a limited number or type of comorbidities, particularly for people with non-KRT-dependent CKD [6,7]. Worse HRQoL has been linked with poorer outcomes, including all-cause mortality, cardiovascular events (and mortality), CKD progression and need for KRT [7,15–18]. In turn, comorbidities can worsen HRQoL and may influence HRQoL more than the CKD itself [7]. For example, in the Renal Impairment In Secondary Care (RIISC) study, comorbidities were associated with a higher risk of impaired HRQoL than kidney function measures [8].

It is therefore important to understand the complex temporal relationship between MLTC and clinically important outcomes in prospective cohorts of people with CKD to identify opportunities for prevention and priorities for management. We therefore aimed to determine the impact of a wide range of comorbidities on mortality, CKD progression and longitudinal HRQoL among people with non-KRT-dependent CKD using data from NURTuRE-CKD, a large prospective cohort study of people with CKD referred to UK nephrology clinics.

## Methods

The main methods for this cohort study have been described elsewhere [19]. In brief, NURTuRE-CKD is a longitudinal study of 2996 people with non-dialysis dependent CKD not in receipt of a kidney transplant recruited from 16 kidney outpatient clinics across the UK. Research Ethics Committee approval for NURTuRE-CKD was granted by South Central-Berkshire Research Ethics

Committee in 2016 (16/SC/0623) and the study abides by the principles of the Declaration of Helsinki. Recruitment took place between 2017 and 2019, first follow up (face to face) between 2018 and 2023, and second follow up by postal questionnaire sent on 10th October 2023 with responses returned by 10th March 2024. Written informed consent was obtained from all participants. NURTuRE-CKD was established to investigate factors that increase the risk of CKD progression, major vascular disease events, and death. The NURTuRE-CKD HRQoL study was initiated to investigate factors associated with HRQoL outcomes in this cohort [19].

Inclusion criteria were: people aged 18 years or over, with an estimated glomerular filtration rate (eGFR) of 15-59 mL/min/1.73 m^2^ or ≥60 mL/min/1.73 m^2^ and a urine albumin to creatinine ratio (uACR) of >30 mg/mmol, who were able to provide written informed consent. They needed to have visited a secondary care nephrology clinic at least once, not have received a solid organ transplant or need kidney replacement therapy (dialysis or transplant) and expected to live for more than a year from enrolment (in the judgment of the local investigators at the recruitment sites). Exclusion criteria were being on chemotherapy for cancer or having experienced acute kidney injury or a major cardiovascular event within the previous three months [19,20]. NURTuRE-CKD was designed as a cohort of people with CKD stages G1-G4 at relatively high risk of adverse outcomes and excluding those with kidney failure and dialysis and transplant patients. However, at recruitment a number of people were already in stage 5 CKD (*n* = 93). Those individuals were therefore excluded from the analyses of the relationship between multiple long-term conditions and CKD progression. For the whole cohort, median eGFR at baseline was 33.8 (IQR 24.0–46.6) mL/min/1.73 m^2^ and among the 2726 participants in whom uACR was measured, 2087 (76.6%) had significant albuminuria.[19]

Sociodemographic, clinical, anthropometric, blood and urine test results were collected at baseline and first follow up. Sociodemographic variables comprised age, sex, educational attainment (categorised as none, secondary level only (General Certificate of Secondary Education (GCSE), National Vocational Qualification (NVQ), or Advanced (A) levels), and higher education (first or higher degree), ethnicity, alcohol use, smoking status, number of medicines and socioeconomic status using the Index of Multiple Deprivation (IMD) which estimates small area level deprivation in the UK. IMD was obtained for all participants in England and adjusted for participants in Wales and Scotland using the method by Abel et al. [21].

Routine laboratory results were available from local laboratories. eGFR and uACR were measured at a central laboratory at baseline. Information on comorbidities at baseline had been collected for a wide range of conditions. These comprised history of abdominal aortic aneurysm, atrial fibrillation, hypertension, ischaemic heart disease (IHD, including history of myocardial infarction or coronary artery bypass graft), heart failure, diabetes (including evidence of diabetes end organ damage, diabetic nephropathy, peripheral neuropathy, foot ulcers, retinopathy, amputation), cerebrovascular disease (including history of stroke or transient ischaemic attack), peripheral vascular disease, asthma, chronic obstructive pulmonary disease (COPD), cancer history, connective tissue and autoimmune disease (including systemic lupus erythematosus, all forms of inflammatory arthritis, scleroderma and vasculitis), dementia (diagnosis and/or dementia drug use), gastric ulcer, and liver disease (mild, moderate or severe).

Further comorbidities were derived from measures used at baseline and first follow up. These included depression and anxiety (defined using the Hospital Anxiety and Depression score (HADS), a score of eight and above in either domain indicating anxiety or depression) [22], cognitive impairment (using the Six-item Cognitive Impairment Test (6CIT), with a score of eight or above indicating cognitive impairment) [23], sarcopenia (defined by the timed up and go test of > 20 s or hand grip strength of < 27 kg in men and < 16 kg in women indicating probable sarcopenia) [24]. Other derived conditions included obesity (defined as BMI ≥30kg/m^2^) [25], hyperuricaemia (defined as urate levels ≥360 umol/l at baseline, anaemia (defined as haemoglobin ≤100 g/L) and pain. The Integrated Palliative care Outcome Score for Renal (IPOS-Renal) Patient Version asks patients to report, for a series of symptoms, including pain, ‘For each symptom, please tick the box that best describes how it has affected you over the past week (Options: not at all (0), slightly (1), moderately (2), severely (3), overwhelmingly (4))’. For this study, pain being present was defined as a score of one (“slightly”) or above [26]. For analyses, comorbidities were categorised into 19 groups: atrial fibrillation, hypertension, IHD, heart failure, diabetes and related complications, cerebrovascular disease, peripheral vascular disease, respiratory disease, cancer history, connective tissue and autoimmune disease, gastric ulcer, liver disease, mental health conditions (anxiety and depression), cognitive impairment, sarcopenia, obesity (BMI ≥30kg/m^2^), hyperuricaemia, anaemia, and pain. MLTC was defined as two or more long-term conditions including CKD based on these 19 groups. The Charlson Comorbidity Index (CCI) was also calculated for each participant by assigning weights for different conditions from a pre-specified list based on their prediction of one- and ten-year mortality [27]. CCI scores have been categorised in some studies to reflect the severity of comorbidity (‘mild’ (CCI score 1–2), ‘moderate’ (CCI score 3–4), and ‘severe’ (CCI score ≥5) [28].

## Outcomes

All-cause mortality was identified at both follow up time points, with latest available mortality data December 31st 2022. CKD progression was defined as progression to kidney failure (the first instance of eGFR <15 mL/min/1.73 m^2^ (sustained for at least 28 days) or initiation of KRT (transplantation or dialysis)) [29]. Outcome data were provided by the UK Renal Registry (UKRR) and registry data extraction was independent of whether a person returned for first or second follow up. HRQoL was defined using the EuroQoL EQ-5D-5L, which consists of a descriptive system and a visual analogue scale. The descriptive system comprises five dimensions: mobility, self-care, usual activities, pain/discomfort and anxiety/depression. Each dimension has five levels: one (no problems), two (slight problems), three (moderate problems), four (severe problems) and five (extreme problems) [30]. The visual analogue scale (VAS) rates from 0 to 100 (where 0 is ‘the worst health you can imagine’ and 100 is ‘the best health you can imagine’) [30]. The five dimensions can be used to derive country-specific index values to obtain a EQ-5D-5L mapped index value. The National Institute for Health and Care Excellence (NICE) has recommended that EQ-5D-5L index values be translated to EQ-5D-3L index values using a method described by Hernandez Alava et al. [31–33]. An EQ-5D-3L value of 1 represents perfect health, 0 death, and negative values health states worse than death [30].

## Statistical analysis

Baseline variables were described using means and standard deviations, median with interquartile range, and frequency counts with percentages. Unadjusted and multivariable shared frailty Cox regression models were used to determine the association between the number of comorbidities and mortality. A non-parametric Kaplan Meier survival curve was used to graphically display the survival probability. Proportional hazards assumptions were checked using Schoenfeld residuals. The association between number of comorbidities and CKD progression was explored using both shared frailty Cox regression models and the Fine and Gray method, due to the competing risks nature of the data.[34] Our results are presented as cause-specific hazard ratios (SHRs) for CKD progression, which describe the relative effect of covariates on the hazard of the event, among those who haven’t experienced the competing risk of death yet. This removes those who experience the competing risk of death from the at risk set for CKD progression when they die. A supporting cumulative incidence plot was also produced to graphically display the probability of CKD progression. Competing risk analysis is appropriate in this situation because it is not possible to experience the CKD progression outcome if the death outcome occurs first.

Adjustment was undertaken for the following potential confounders: age, sex, ethnicity, socioeconomic status, alcohol use, educational status, smoking status, number of medicines, eGFR, uACR. These were based on clinical input and consistent with baseline analyses [19,20]. Region of recruitment was added as a random effect to account for clustering. Stata SE 19.5 was used for analyses. Those who had not reached an outcome event were censored at the last UKRR follow up date (December 31, 2022), except for the small number who had a transplant in 2023. Unadjusted and multivariable mixed effects linear regression was used to determine associations between the number and type of comorbidity and HRQoL (using EQ-5D-3L index value) at baseline and both follow up time points.

## Patient and public involvement

The NURTuRE-CKD HRQoL study was supported by a four-person patient advisory group. Members of the group were involved in the design of the questionnaire for the second follow up point and discussion and interpretation of the findings.

## Results

**Figure 1.**
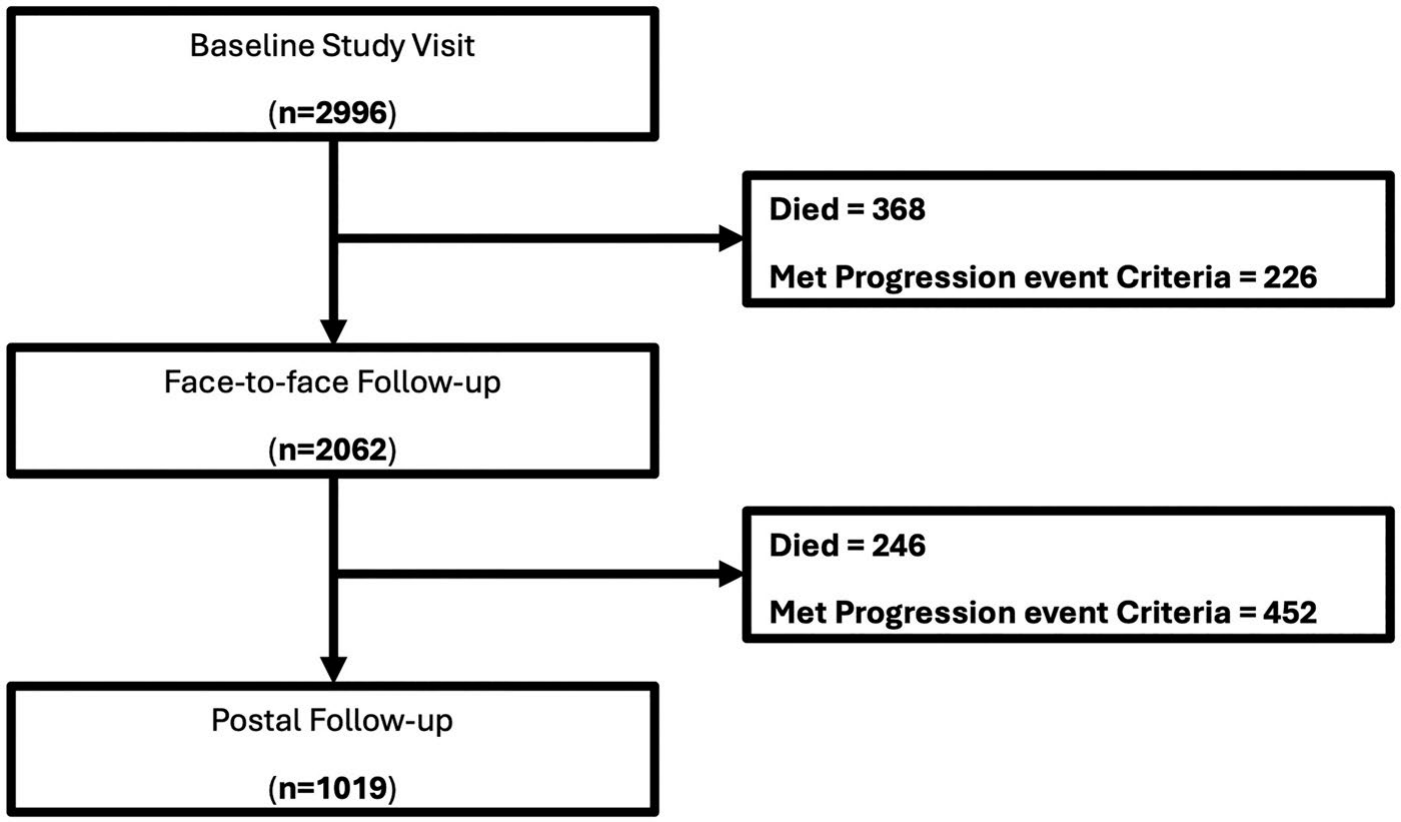
Flow chart of participant data collection. Footnote to Figure 1: Progression and mortality data were collected from registry information and so were independent of follow-up attendance or questionnaire completion.

**Figure 2.**
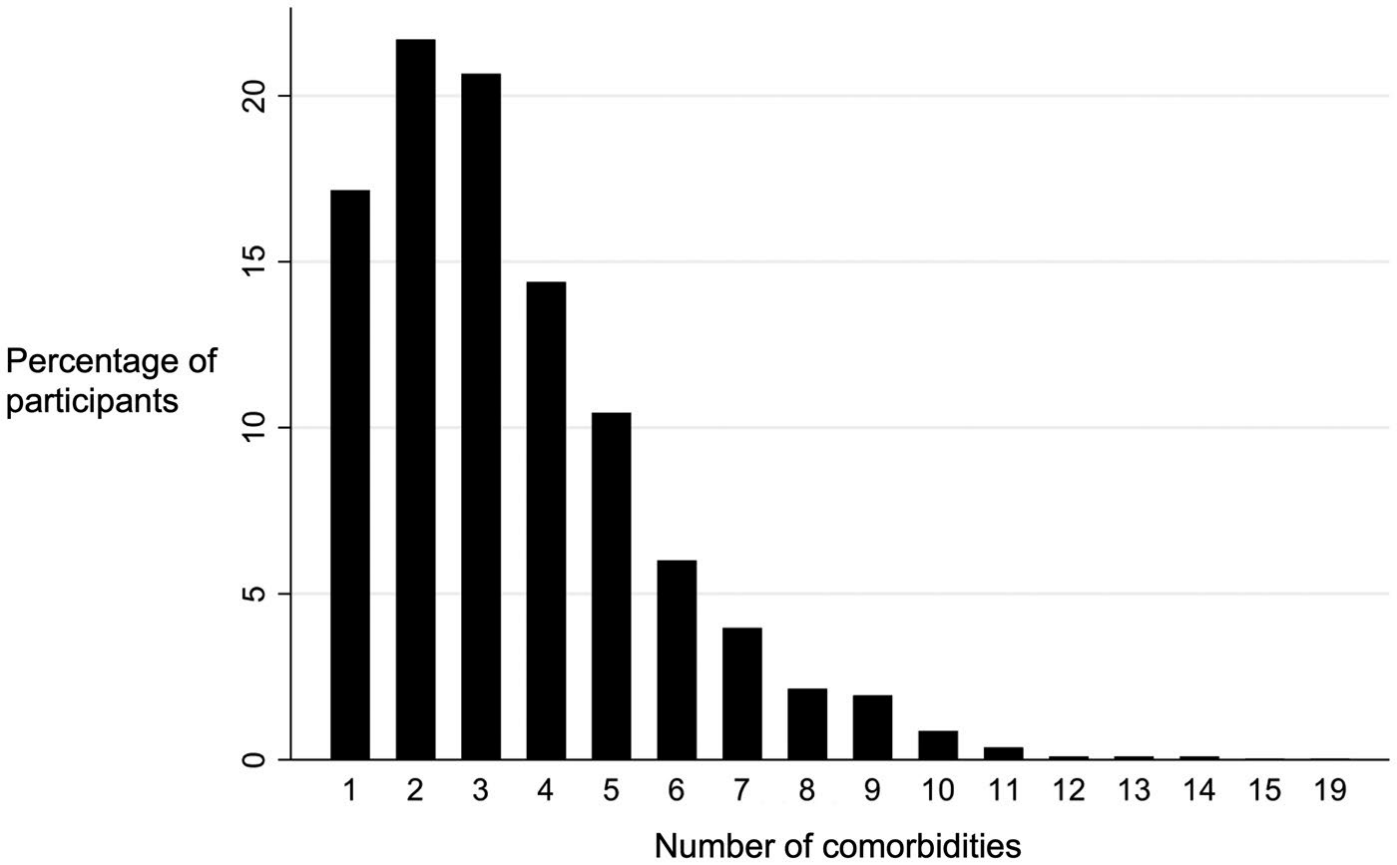
Distribution of the number of comorbidities among NURTuRE-CKD participants at baseline.

Of 2996 people recruited at baseline, 2062 (68.8%) completed first follow up and 1019 (34.0%) responded to the subsequent follow up postal questionnaire (Figure 1). Median (IQR) time between baseline and follow up was 1.42 years (1.13, 2.25, first) and 5.25 years (4.89, 5.92, second). Median (IQR) time to the mortality outcome was 2.06 years (1.16 to 3.05 years) and to CKD progression was 1.95 years (1.01 to 3.04 years). At baseline, the mean age was 63 years, 1243 (41%) were female, 2613 (87%) were white, and 862 (29%) had no educational qualifications. Mean eGFR was 37.3 mL/min/1.73m^2^ (Table 1). The number of comorbidities present at baseline ranged from one to 19, (median 3, interquartile range (IQR) 2 to 5). All participants had at least one other comorbidity in addition to their CKD with 650 (22%), 619 (21%) and 431 (14%) having two, three and four comorbidities respectively, and 782 (26%) having five or more (Figure 2). The median CCI score was 3.00 (IQR 2.00 to 5.00) and the commonest comorbidities comprised hypertension 2503 (84%), pain 1851 (62%), and obesity (BMI ≥30kg/m^2^) 1201 (41%) (Table 2). By the first follow up, 285 (14%), 226 (11%.), 115 (6%), 98 (5%) and 82 (4%) people reported new pain, mental health conditions, cognitive impairment, obesity and anaemia, respectively (Table 2). By the end of follow up (December 31, 2022) 614/2996 (20.5%) people died, and 678/2893 (23%) progressed to kidney failure.

**Table 1.** Baseline characteristics of NURTuRE-CKD participants unless otherwise stated, all variables are presented as number and percentage.

		One comorbidity *N* = 514	Two Comorbidities *N* = 650	Three comorbidities *N* = 619	Four comorbidities *N* = 431	Five or more comorbidities *N* = 782	Total *N* = 2996
**Age**	(Mean +/- SD)	56.18 +/– 16.05	58.97 +/– 15.51	63.62 +/– 14.09	65.01 +/– 13.16	67.75 +/– 12.01	62.61 +/– 14.75
**Sex**	Female	218 (42.4)	273 (42.0)	233 (37.6)	176 (40.8)	343 (43.9)	1243 (41.4)
	Male	296 (57.6)	377 (58.0)	386 (62.4)	255 (59.2)	439 (56.1)	1753 (58.5)
**Ethnicity**	Asian	53 (10.3)	54 (8.3)	43 (7.0)	20 (4.6)	30 (3.8)	200 (6.7)
	Black	19 (3.7)	25 (3.9)	26 (4.2)	10 (2.3)	11 (1.4)	91 (3.0)
	Mixed	7 (1.4)	11 (1.7)	9 (1.5)	1 (0.2)	6 (0.8)	34 (1.1)
	Other	18 (3.5)	14 (2.2)	10 (1.6)	7 (1.6)	4 (0.5)	53 (1.8)
	White	416 (81.0)	545 (84.0)	528 (85.7)	393 (91.2)	731 (93.5)	2613 (87.4)
**Socioeconomic status (by quintile of Index of Multiple Deprivation (IMD)**	1 (most deprived)	119 (23.2)	142 (21.9)	147 (23.8)	94 (22.0)	144 (18.4)	646 (21.6)
	2	114 (22.2)	132 (20.3)	129 (20.9)	88 (20.6)	154 (19.7)	617 (20.6)
	3	88 (17.2)	121 (18.6)	110 (17.8)	90 (21.0)	148 (18.9)	557 (18.6)
	4	85 (16.6)	124 (19.1)	102 (16.5)	81 (18.9)	158 (20.2)	550 (18.3)
	5 (least deprived)	107 (20.9)	130 (20.0)	130 (21.0)	75 (17.5)	178 (22.8)	620 (20.7)
**Educational attainment**	None	119 (23.1)	157 (24.2)	189 (30.5)	127 (29.5)	270 (34.5)	862 (28.8)
	GCSE/NVQ/A levels*	226 (44.0)	299 (46.0)	277 (44.8)	195 (45.2)	354 (45.3)	1351 (45.1)
	Higher education	169 (32.9)	194 (29.9)	153 (24.7)	109 (25.3)	158 (20.2)	783 (26.1)
**Smoking status**	None	287 (57.2)	345 (54.2)	324 (53.0)	204 (47.8)	323 (41.5)	1483 (50.2)
	Ex smoker	172 (34.3)	239 (37.5)	232 (38.0)	182 (42.6)	384 (49.4)	1209 (40.9)
	Current smoker	43 (8.6)	53 (8.3)	55 (9.0)	41 (9.6)	71 (9.1)	263 (8.9)
**Alcohol intake (any amount)**	No	224 (44.8)	275 (43.6)	269 (44.5)	188 (44.1)	414 (53.4)	1370 (46.7)
	Yes	276 (55.2)	356 (56.4)	336 (55.5)	238 (55.9)	361 (46.6)	1567 (53.4)
**Number of medications**	(Mean +/- SD)	4.63 +/– 2.87	6.34 +/– 3.57	7.54 +/– 3.94	8.10 +/– 3.80	11.07 +/– 4.74	7.78 +/– 4.51
**Health-related quality of life**	EQ-5D-3L mapped index (median, IQR)	0.89 (0.75, 0.99)	0.86 (0.71, 0.99)	0.80 (0.66, 0.99)	0.75 (0.61, 0.99)	0.66 (0.43, 0.79)	0.78 (0.63, 0.99)
	EQ-5D-5L health rating (visual analogue scale) (mean ± SD)	79.74 +/– 16.21	76.57 +/– 17.87	74.72 +/– 17.55	70.51 +/– 20.91	64.47 +/– 21.25	72.57 +/– 19.77
	Mobility issues	131 (25.5)	218 (33.5)	297 (48.0)	237 (55.0)	582 (74.4)	1465 (48.9)
	Self-care	26 (5.1)	78 (12.0)	77 (12.4)	87 (20.2)	280 (35.8)	548 (18.3)
	Usual activities	119 (23.2)	214 (32.9)	266 (43.0)	209 (48.5)	516 (66.0)	1324 (44.2)
	Pain	220 (42.8)	332 (51.1)	346 (55.9)	275 (63.8)	600 (76.7)	1773 (59.2)
	Anxiety and depression	121 (23.5)	182 (28.0)	209 (33.8)	154 (35.7)	348 (44.5)	1014 (33.9)
**Charlson comorbidity index (mean, SD)**	(Mean +/- SD)	1.75 +/– 1.45	2.54 +/– 1.77	3.44 +/– 1.89	3.88 +/– 1.95	5.09 +/– 2.07	3.45 +/– 2.20
**eGFR mL/min/1.73 m2**		41.5 +/– 20.7	38.3 +/– 18.6	36.7 +/– 17.2	37.2 +/– 18.2	34.2 +/– 14.7	37.3 +/– 17.9
**Urine albumin creatinine ratio (uACR) mg/g**		95.7 +/– 515.6	92.9 +/– 149.8	85.3 +/– 143.4	86.4 +/– 147.7	90.3 +/– 158.7	90.2 +/– 254.7

*General Certificate of Secondary Education, National Vocational Qualification, Advance level certificate.

**Table 2.** Prevalence of comorbidities at baseline and first follow up.

Comorbidity	Baseline Total *n* = 2996 Number (%)	First follow up Total *n* = 2062 Number (%)	New conditions reported at first follow up Number (% of those at first follow up)
Hypertension	2503 (84.4)	1739 (84.3)	12 (0.6)
Pain (IPOS)	1851 (62.4)	1301 (64.1)	285 (13.8)
Obesity (BMI ≥30kg/m^2^)	1201 (40.5)	795 (38.6)	98 (4.8)
Hyperuricemia (Urate levels ≥360 g/litre)*	1031 (34.7)		
Mental health conditions (anxiety and depression)	937 (31.6)	669 (32.5)	226 (10.9)
Diabetes, diabetes nephropathy, diabetes retinopathy, diabetes end organ damage and peripheral neuropathy	930 (31.4)	586 (28.9)	6 (0.3)
Sarcopenia (a timed up and go test of >20 s and a hand grip strength of <27 kg in men and < 16 kg in women indicating probable sarcopenia*	906 (30.5)		
Connective tissue and autoimmune disease	770 (26.0)	283 (13.7)	6 (0.3)
Respiratory disease (COPD and asthma)	504 (17.0)	171 (8.3)	7 (0.3)
Cancer history	444 (15.0)	315 (15.3)	8 (0.4)
Ischemic heart disease (IHD)	326 (11.0)	119 (6.6)	1 (0.05)
Atrial fibrillation	293 (9.9)	196 (9.5)	7 (0.3)
Cerebrovascular disease	258 (8.7)	99 (4.8)	4 (0.2)
Cognitive impairment (6-CIT)	247 (8.3)	179 (8.6)	115 (5.6)
Heart failure	155 (5.2)	366 (17.8)	20 (1.0)
Anaemia (Hb levels ≤100 g/litre)	152 (5.0)	99 (4.8)	82 (4.0)
Liver disease	146 (4.9)	115 (5.6)	4 (0.2)
Peripheral vascular disease (PVD)	125 (4.2)	77 (3.7)	5 (0.2)
Gastric ulcer	97 (3.3)	73 (3.5)	4 (0.2)

*Data not available at first follow up.

## Mortality and CKD progression

**Figure 3.**
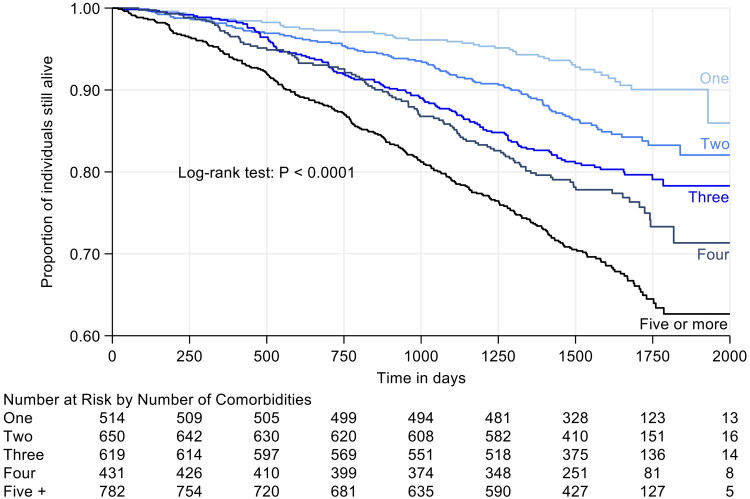
Kaplan Meier plot of survival by number of comorbidities.

A clear relationship was observed between the number of comorbidities and mortality in unadjusted and multivariable models. Compared to people with one comorbidity at baseline, adjusted hazard ratios (HR) for those with two, three, four and five or more were 1.37 (0.93 to 2.03),1.45 (0.99 to 2.13), 1.54 (1.03 to 2.29) and 1.80 (1.23 to 2.63) respectively (Table 3, Figure 3, Supplementary Table 1). There was no significant evidence against the proportional hazards assumption (global p value = 0.2622). Compared to people with one comorbidity at baseline, a greater number of comorbidities was not associated with CKD progression to kidney failure (HRs 1.14 (0.88 to 1.47), 0.84 (0.63 to 1.11), 0.79 (0.57 to 1.08) and 0.91 (0.67 to 1.23) for two, three, four and five or more comorbidities, respectively. (Supplementary Table 2, Supplementary Figure 1).

**Table 3.** Association between number of comorbidities at baseline and mortality.

		Unadjusted			Multivariable^a^		
		Hazard ratio	95% Confidence interval	*p* value	Hazard ratio	95% Confidence interval	*p* value
**Comorbidities**	1(reference)						
	2	1.81	(1.26 to 2.59)	0.001	1.37	(0.93 to 2.03)	0.110
	3	2.48	(1.75 to 3.51)	<0.001	1.45	(0.99 to 2.13)	0.056
	4	3.04	(2.13 to 4.35)	<0.001	1.54	(1.03 to 2.29)	0.035
	≥5	4.61	(3.33 to 6.38)	<0.001	1.80	(1.23 to 2.63)	0.002
**Age**		1.07	(1.07 to 1.08)	<0.001	1.06	(1.05 to 1.07)	<0.001
**Sex (vs. Male)**	Female	0.65	(0.55 to 0.77)	<0.001	0.77	(0.63 to 0.94)	0.009
**Ethnicity (vs. White)**	Asian	0.43	(0.27 to 0.68)	<0.001	0.81	(0.49 to 1.33)	0.397
	Black	0.94	(0.59 to 1.51)	0.804	1.10	(0.65 to 1.84)	0.724
	Mixed	0.49	(0.18 to 1.32)	0.159	0.53	(0.13 to 2.18)	0.381
	Other	0.38	(0.14 to 1.02)	0.055	0.54	(0.18 to 1.67)	0.288
**Socioeconomic status (by quintile of Index of Multiple Deprivation (IMD**)							
	2	1.26	(0.97 to 1.64)	0.082	1.20	(0.91 to 1.59)	0.204
	3	1.45	(1.11 to 1.88)	0.006	1.28	(0.96 to 1.70)	0.094
	4	1.61	(1.24 to 2.08)	<0.001	1.60	(1.21 to 2.13)	0.001
	5	1.39	(1.07 to 1.79)	0.014	1.49	(1.10 to 2.00)	0.009
**Educational attainment (vs. none)**	GCSE/NVQ/A level^b^	0.53	(0.44 to 0.63)	<0.001	0.95	(0.78 to 1.17)	0.630
	Higher education	0.43	(0.34 to 0.54)	<0.001	0.93	(0.72 to 1.21)	0.580
**Smoking (vs None)**	Ex smoker	1.80	(1.52 to 2.13)	<0.001	1.27	(1.05 to 1.53)	0.014
	Current smoker	1.51	(1.14 to 2.01)	0.005	1.80	(1.30 to 2.49)	<0.001
**Alcohol use (vs No)**		0.75	(0.64 to 0.88)	<0.001	0.89	(0.74 to 1.07)	0.220
**Number of medications**		1.10	(1.08 to 1.11)	<0.001	1.05	(1.02 to 1.07)	<0.001
**eGFR mL/min/1.73 m^2^**		0.96	(0.96 to 0.97)	<0.001	0.98	(0.97 to 0.99)	<0.001
**uACR mg/g**		1.00	(1.00 to 1.00)	0.119	1.00	(1.00 to 1.00)	0.032

^a^Model adjusted for age, sex, ethnicity, socioeconomic status, alcohol use, educational status, smoking status, number of medicines, eGFR, uACR. Region of recruitment was added as a random effect.

^b^General Certificate of Secondary Education, National Vocational Qualification, Advance level certificate.

## HRQoL at first and second follow up points

Complete HRQoL (EQ-5D-5L) data were available for 2054/2996 (68.5%) participants at first follow up and 1019/2996 (34.0%) at all three time points. The mean EQ-5D-3L mapped index value at baseline, first and second follow up was 0.75 (± 0.25), 0.73 (± 0.26) and 0.66 (± 0.28) respectively. At baseline, 2201 (74.4%) had problems in at least one EQ-5D-5L dimension as previously reported [20]. At first follow up, 1595 (78%) people reported problems in at least one EQ-5D-5L dimension. By the second follow up, this had increased to 871 (85%) reporting problems in at least one dimension.

## Associations with HRQoL

Unadjusted analyses showed that greater number of baseline comorbidities was associated with worse HRQoL at all three time points (Table 4 and Supplementary Tables 3 and 4). At first follow up the association increased progressively with greater number of comorbidities until seven comorbidities, when the trend levelled off somewhat (Supplementary Figure 2). A similar pattern was observed for multivariable analysis (Table 4 and Supplementary Tables 3 and 4). Other variables associated with worse HRQoL were being female, greater socioeconomic deprivation, lower educational attainment, ex or current smoking, not drinking alcohol, and taking more medications (Table 4).

**Table 4.** Association between the number of baseline comorbidities and health-related quality of life at first follow up.

		Unadjusted			Multivariable^a^		
		Coefficient	95% Confidence interval	*p* value	Coefficient	95% Confidence interval	*p* value
**Number of comorbidities (vs. 1)**	2	−0.02	(-0.05 to 0.01)	0.259	−0.00	(−0.03 to 0.03)	0.949
	3	−0.06	(-0.09 to −0.02)	0.001	−0.01	(−0.04 to 0.02)	0.553
	4	−0.11	(-0.15 to −0.08)	<0.001	−0.05	(−0.09 to −0.01)	0.007
	≥5	−0.23	(-0.26 to −0.20)	<0.001	−0.11	(–0.14 to −0.07)	<0.001
**Age**		−0.002	(-0.00 to −0.00)	<0.001	−0.00	(–0.00 to 0.00)	0.640
**Sex (vs. Male)**	Female	−0.07	(-0.09 to −0.05)	<0.001	−0.06	(–0.08 to −0.04)	<0.001
**Ethnicity (vs. White)**	Asian	0.02	(-0.03 to 0.06)	0.512	0.01	(–0.04 to 0.05)	0.781
	Black	0.02	(-0.05 to 0.09)	0.616	0.02	(–0.04 to 0.09)	0.491
	Mixed	0.05	(-0.06 to 0.16)	0.350	0.04	(–0.06 to 0.14)	0.452
	Other	0.05	(-0.04 to 0.14)	0.284	0.05	(–0.03 to 0.13)	0.243
**Socioeconomic status (by quintile of Index of Multiple Deprivation (IMD**)	1 most deprived (reference)						
	2	0.02	(-0.02 to 0.05)	0.341	0.02	(–0.01 to 0.05)	0.112
	3	−0.03	(-0.07 to 0.00)	0.058	−0.01	(–0.04 to 0.02)	0.501
	4	−0.07	(-0.11 to −0.04)	<0.001	−0.05	(–0.08 to −0.02)	0.002
	5	−0.11	(-0.14 to −0.07)	<0.001	−0.05	(–0.08 to −0.02)	0.002
**Educational attainment (vs. none)**	GCSE/NVQ/A level^b^	0.07	(0.05 to 0.10)	<0.001	0.03	(0.00 to 0.06)	0.034
	Higher education	0.14	(0.11 to 0.17)	<0.001	0.05	(0.03 to 0.07)	0.002
**Smoking (vs None)**	Ex smoker	−0.06	(-0.08 to −0.04)	<0.001	−0.03	(–0.05 to −0.01)	0.007
	Current smoker	−0.11	(-0.15 to −0.07)	<0.001	−0.07	(–0.11 to −0.03)	<0.001
**Alcohol use (vs No**	Yes	0.11	(0.09 to 0.14)	<0.001	0.06	(0.03 to 0.08)	<0.001
**Number of medications**		−0.02	(-0.02 to −0.02)	<0.001	−0.01	(–0.02 to −0.01)	<0.001
**eGFR mL/min/1.73 m^2^**		0.001	(0.001 to 0.002)	<0.001	−0.00	(–0.00 to 0.00)	0.788
**uACR mg/g**		−0.00	(-0.00 to 0.00)	0.550	−0.00	(–0.00 to 0.00)	0.743

^a^Model adjusted for age, sex, ethnicity, socioeconomic status, alcohol use, educational status, smoking status, number of medicines, eGFR, uACR. Region of recruitment was added as a random effect (random intercepts).

^b^General Certificate of Secondary Education, National Vocational Qualification, Advance level certificate.

In terms of specific comorbidities, peripheral vascular disease, cerebrovascular diseases, respiratory disease, gastrointestinal disease, diabetes and related complications, heart failure, atrial fibrillation, obesity, pain, sarcopenia, anaemia, cancer, ischaemic heart diseases, mental health conditions (anxiety and depression), and cognitive impairment were all associated with worse HRQoL in the unadjusted analyses (Table 5). In multivariable analysis, most remained associated with worse HRQoL. At second follow up, peripheral vascular disease, obesity, pain, sarcopenia, and mental health conditions remained associated with worse HRQoL (Supplementary Table 5).

**Table 5.** Association between specific baseline comorbidities and health-related quality of life at first follow up.

		Unadjusted			Multivariable^a^			Multivariable^b^		
		Coefficient	95% confidence interval	*p* value	Coefficient	95% confidence interval	*p* value	Coefficient	95% confidence interval	*p* value
**Comorbidities**	Peripheral vascular disease (PVD)	−0.17	(−0.21 to −0.12)	<0.001	−0.17	(−0.21 to −0.12)	<0.001	−0.06	(−0.10 to −0.02)	<0.001
	Cerebrovascular disease	−0.14	(−0.18 to −0.09)	<0.001	−0.12	(−0.16 to −0.08)	<0.001	−0.04	(−0.08 to −0.01)	<0.001
	Respiratory	−0.07	(−0.10 to −0.04)	<0.001	−0.07	(−0.10 to −0.04)	<0.001	−0.00	(−0.03 to 0.03)	0.84
	Gastrointestinal	−0.06	(−0.12 to −0.00)	<0.001	−0.05	(−0.11 to 0.01)	0.11	0.02	(−0.04 to 0.08)	0.54
	Diabetes and complications	−0.12	(−0.14 to −0.09)	<0.001	−0.12	(−0.14 to −0.09)	<0.001	−0.02	(−0.04 to 0.00)	0.08
	Hypertension	0.01	(−0.02 to 0.05)	0.65	0.004	(−0.02 to 0.03)	0.77	0.03	(0.01 to 0.06)	<0.001
	Heart failure	−0.11	(−0.16 to −0.05)	<0.001	−0.08	(−0.13 to −0.03)	<0.001	−0.01	(−0.07 to 0.04)	0.65
	AF	−0.09	(−0.12 to −0.05)	<0.001	−0.07	(−0.11 to −0.03)	<0.001	−0.04	(−0.07 to −0.01)	<0.001
	Obesity	−0.09	(−0.12 to −0.07)	<0.001	−0.09	(−0.11 to −0.07)	<0.001	−0.05	(−0.07 to −0.03)	<0.001
	Pain	−0.18	(−0.20 to −0.16)	<0.001	−0.17	(−0.19 to −0.14)	<0.001	−0.09	(−0.12 to −0.07)	<0.001
	Sarcopenia	−0.15	(−0.17 to −0.12)	<0.001	−0.13	(−0.15 to −0.10)	<0.001	−0.04	(−0.07 to −0.02)	<0.001
	Connective tissue disease + Autoimmune disease	0.01	(−0.01 to 0.03)	0.43	0.002	(−0.02 to 0.02)	0.86	−0.02	(−0.04 to 0.01)	0.16
	Anaemia	−0.10	(−0.16 to −0.04)	<0.001	−0.09	(−0.15 to −0.03)	<0.001	−0.01	(−0.06 to 0.03)	0.71
	Cancer	−0.05	(−0.08 to −0.02)	<0.001	−0.03	(−0.06 to 0.00)	0.06	−0.03	(−0.06 to −0.01)	<0.001
	Liver disease	−0.04	(−0.09 to 0.01)	0.10	−0.04	(−0.08 to 0.01)	0.14	−0.00	(−0.0 to 0.04)	0.92
	Hyperuricaemia	−0.01	(−0.03 to 0.02)	0.53	−0.01	(−0.04 to 0.00)	0.24	−0.00	(−0.03 to 0.01)	0.46
	Mental health conditions	−0.20	(−0.22 to −0.18)	<0.001	−0.21	(−0.23 to −0.18)	<0.001	−0.13	(−0.15 to −0.11)	<0.001
	Cognitive impairment	−0.09	(−0.14 to −0.05)	<0.001	−0.09	(−0.13 to −0.04)	<0.001	−0.03	(−0.07 to 0.02)	0.23
	Ischemic heart disease (IHD)	−0.09	(−0.13 to −0.05)	<0.001	−0.09	(−0.12 to −0.05)	<0.001	−0.01	(−0.05 to −0.03)	0.71
**Age**		−0.002	(−0.00 to −0.001)	<0.001	−0.003	(−0.03 to −0.00)	<0.001	−0.00	(−0.00 to 0.00)	0.53
**Sex (vs. male)**	Female	−0.07	(−0.09 to −0.05)	<0.001	−0.08	(−0.10 to −0.06)	<0.001	−0.04	(−0.05 to −0.00)	<0.001
**Ethnicity (vs. white)**	Asian	0.02	(−0.03 to 0.06)	0.51	−0.02	(−0.06 to 0.09)	0.51	0.03	(−0.01 to 0.07)	0.09
	Black	0.02	(−0.05 to 0.09)	0.61	0.00	(−0.06 to 0.16)	0.99	0.04	(−0.01 to 0.10)	0.14
	Other	0.05	(−0.04 to 0.14)	0.28	0.04	(−0.04 to 0.15)	0.41	0.06	(−0.02 to 0.13)	0.15
	Mixed	0.05	(−0.06 to 0.16)	0.35	0.03	(−0.03 to 0.06)	0.52	−0.04	(−0.06 to 0.13)	0.46
**Socioeconomic status (by quintile of Index of Multiple Deprivation (IMD, vs 1, most deprived)**)										
	1 (most deprived)									
	2	0.04	(0.001 to 0.07)	<0.001	0.03	(0.00 to 0.07)	0.05	−0.01	(−0.04 to 0.02)	0.61
	3	0.08	(0.04 to 0.11)	<0.001	0.08	(0.04 to 0.11)	<0.001	0.02	(−0.01 to 0.05)	0.26
	4	0.12	(0.09 to 0.16)	<0.001	0.13	(0.10 to 0.17)	<0.001	0.05	(0.02 to 0.08)	<0.001
	5	0.11	(0.07 − 0.14)	<0.001	0.12	(0.08 to 0.15)	<0.001	0.02	(−0.01 to 0.05)	0.12
**Educational attainment (vs. none)**	GCSE/NVQ/A level^c^	0.07	(0.05 to 0.10)	<0.001	0.06	(0.03 to 0.09)	<0.001	<0.001	(−0.01 to 0.05)	0.47
	Higher education	0.14	(0.11 to 0.17)	<0.001	0.12	(0.09 to 0.15)	<0.001	<0.001	(−0.01 to 0.06)	0.27
**Smoking (vs None**	Ex smoker	−0.06	(−0.15 to −0.07)	<0.001	−0.05	(−0.07 to −0.02)	<0.001	−0.02	(−0.05 to −0.00)	<0.001
	Current smoker	−0.11	(−0.08 to − 0.04)	<0.001	−0.12	(−0.17 to −0.09)	<0.001	−0.05	(−0.09 to −0.01)	<0.001
**Alcohol use (vs None)**	Yes	0.11	(0.09 to 0.13)	<0.001	0.10	(0.08 to 0.12)	<0.001	0.04	(0.01 to 0.06)	<0.001
**Number of medications**		−0.02	(−0.02 to − 0.01)	<0.001	−0.02	(−0.02 to −0.01)	<0.001	−0.01	(−0.01 to −0.00)	<0.001
**eGFR mL/min/1.73 m^2^**		0.001	(0.00 to 0.002)	<0.001	0.00	(0.00 to 0.001)	<0.001	<0.001	(−0.00 to 0.00)	0.83
**uACR mg/g**		−0.00	(−0.00 to 0.00)	0.36	−0.00	(−0.00 to −0.00	0.03	<0.001	(0.89 to 1.05)	0.36

^a^Model adjusted for age and sex.

^b^Model adjusted for age, sex, ethnicity, socioeconomic status, alcohol use, educational status, smoking status, number of medicines, eGFR, uACR. Region of recruitment was added as a random effect (random intercepts).

^c^General Certificate of Secondary Education, National Vocational Qualification, Advance level certificate.

Those who returned for first follow up were more likely to be younger, to live in more socioeconomically deprived areas, to have better educational attainment, to be non-smokers, to drink alcohol, to take more medications, and to have higher eGFR and lower uACR than those who did not. Those who returned the questionnaire were more likely to be of a white ethnicity, live in more socioeconomically deprived areas, have better educational attainment, be non-smokers, drink alcohol, take less medications, have a lower number of comorbidities and have higher eGFR and lower uACR than those who did not (Supplementary Tables 6 and 7).

## Discussion

In this large cohort of people referred to nephrology centres with non-KRT-dependent CKD, all participants had at least one comorbidity at baseline, meeting the commonly used definition for MLTC. MLTC progressed over time, with the commonest conditions to develop between baseline and first follow up being pain, mental health conditions (anxiety and depression), cognitive impairment, obesity and anaemia. A greater number of comorbidities was a risk factor for higher all-cause mortality but not CKD progression to kidney failure. Both greater number and certain types of comorbidities such as peripheral vascular disease, cardiovascular disease, pain, sarcopenia, obesity and mental health conditions were strongly associated with worse HRQoL at baseline and over time, with risk increasing progressively as the number of comorbidities increased. This suggests that efforts to prevent the accumulation of comorbid conditions, in particular pain, mental health conditions, anaemia and obesity, may be effective in improving survival and preserving HRQoL, though we are unable to infer any causal relationship of such interventions from this observational study. Nevertheless, the associations identified highlight the importance of holistic care, rather than solely a narrow focus on preserving kidney function.

Our findings build on previous research. The median CCI score was 3 (IQR 2 to 5) comparable to the median of 3 (IQR 1 to 5) found in the RIISC study, a similar prospective single-centre cohort of people with high-risk CKD [8]. Even in studies of milder CKD in primary care, a strong association with MLTC was reported. A UK primary care-based cohort study found that only 4% of people with CKD were living without at least one comorbidity [2] while the Kidney Early Evaluation Program (KEEP) study reported a prevalence of 40% for two or more comorbidities [35]. Hypertension, ischaemic heart disease, diabetes, pain, anaemia, thyroid conditions and mental health conditions are common in people with CKD [2,9,36,37]. The RIISC study also associated comorbidities with greater mortality risk [8]. In contrast to our findings, a Taiwanese study exploring the impact of number of comorbidities on outcomes among 1463 people with CKD stage 3-5 identified an association with initiation of long-term dialysis (patients with at least three comorbidities at baseline started dialysis earlier than those without) [14].

Consistent with our findings on HRQoL,, analyses from the Renal Risk in Derby cohort identified a progressive decrease in HRQoL with an increase in comorbidities, with risk of worse HRQoL doubling for three or more comorbidities compared with none or one [37]. A prospective study of HRQoL in the AusDiab study also found the type of comorbidity to be important, with obesity, diabetes, hypertension, cardiovascular diseases, anaemia and hyperuricaemia all associated with worse HRQoL [38]. Comorbidities may therefore be of more immediate concern than kidney function for many people with CKD.

A greater understanding of MLTC and its progression in the context of CKD is clinically relevant because some comorbid conditions may be preventable or treatable. Obesity is common among patients with CKD and is known to be associated with worse HRQoL [20,38]. A reduction in weight and BMI can improve HRQOL, reduce albuminuria and, in some cases, improve kidney function [3,39,40]. This supports greater use of lifestyle interventions and weight-loss management with medicines or bariatric surgery for people with CKD and obesity, as well as wider public health measures to reduce obesity incidence [3]. Dietary interventions (such as DASH and Mediterranean diets) and physical activity play an important role in CKD and its common comorbidities [41]. There is evidence of impact on both prevention and progression of CKD as well as reducing risk of cardiovascular disease and, for physical activity, of improving mental health-related quality of life [41–43].

Other studies have also shown mental health conditions and pain among people with CKD to be associated with worse HRQoL [5,6,37]. Pain management is complex, and pain medications may also have side effects that adversely impact HRQoL. There is therefore a need to identify interventions that can safely improve pain and HRQOL in people living with CKD. The aetiology of mental health conditions and their relationship to CKD and other long-term conditions is complex. Our observations suggest that interventions to prevent or treat them may also improve HRQoL, but evidence on this is currently mixed, including interventions for depression [44]. Sarcopenia is a common and important comorbidity in people with CKD and is related to multiple factors including poor nutrition, physical inactivity and chronic inflammation [45]. It has been associated with worse HRQoL as well as increased fracture risk, hospitalisation and mortality among people with CKD [42]. Importantly, it is potentially reversible with nutritional support, exercise and treatment of inflammation. Anaemia is recognised as a direct consequence of CKD and multiple treatment options are available including iron supplementation, recombinant erythropoietin and hypoxia-inducible factor prolyl hydroxylase inhibitors (HIF-PHI).

A recent qualitative evidence synthesis of 46 studies among people with multiple long-term conditions identified that the patient experience of living with MLTC is multifaceted and complex, encompassing eight themes of ‘work’ [46]. These themes (learning and adapting; accumulation and complexity; symptoms; emotions; investigation and monitoring; health service and administration; medication; and finance) can act together to create cumulative burden for people, with co-existing mental and physical comorbidities presenting particular challenges. Given that all NURTuRE-CKD participants had MLTC, with high prevalence of pain, obesity and mental health conditions, this is highly relevant for the population of people referred to secondary care with CKD. Comorbidities that have a impact on symptoms such as pain, low mood and anxiety may be particularly important for HRQoL. The review identified that navigating the health system and people’s individual context influenced the challenges of living with MLTC at least as much as the specific combination of long-term conditions experienced. Importantly, much of this work of living with MLTC may not be apparent to healthcare workers. Given the high prevalence of MLTC in people with CKD this ‘work’ is likely to be an important factor in the association shown between additional long-term conditions and worsening HRQoL and should be considered in both clinical encounters and in the organisation of health services [47,48].

Our study has several strengths. A unique strength is the combination of longitudinal data on comorbid conditions, mortality, CKD progression and HRQoL. Analysing data from a large cohort of people referred to secondary care with CKD across several UK regions, incorporation of many comorbidities, assessing HRQoL at three time points using a standardised measure, and consideration of a large number of relevant confounding factors, with variables defined using standardised and reproducible methods strengthen the generalisability of our findings. However, there are some limitations. It was not possible to adjust for all potential confounders that could affect HRQoL among people with CKD. The EQ-5D-5L has known limitations; for example, a cross-sectional analysis in a general population found the self-care dimension of the EQ-5D-5L to show a substantial ceiling effect relating to those reporting ‘no problems’ [49]. Defining depression and anxiety by HADS score and cognitive impairment by 6CIT score comes with the caveat that these are screening measures rather than diagnostic tests. Defining pain as a comorbidity using the IPOS-renal score of one (“slightly”) or above arguably sets a low threshold. However, the prevalence of pain in this cohort (62.4% at baseline) was in line with other studies [50]. It was also likely that our results were affected by survivor bias, as there were differences in the characteristics of those who attended first follow up and who returned the questionnaire, compared to those who did not. Around 1500 people died or were lost to follow up, potentially leading to selection bias. We have previously demonstrated that those not attending follow up had worse HRQoL at baseline [51].

We are unable to draw definitive conclusions about the lack of association between comorbidities and progression to kidney failure beyond this specific cohort and length of follow up. The study was potentially still underpowered for the CKD progression outcome due to limited follow up time, and some residual confounding may also have contributed to the lack of association with CKD progression being identified. Finally, our study population was comprised of a majority of white, older participants and the results may therefore not be applicable to populations from other regions with a different demographic composition.

## Conclusion

All participants in this referred population of people with CKD had MLTC. A greater number of comorbidities was a risk factor for higher all-cause mortality and worse longitudinal HRQoL but not CKD progression to kidney failure in this cohort. Specific comorbidities including peripheral vascular disease, cardiovascular disease, atrial fibrillation, obesity, pain, sarcopenia, mental health conditions (anxiety and depression) and cancer, were associated with worse longitudinal HRQoL. Interventions to prevent people with CKD from developing additional conditions and holistic care of people with CKD should be prioritised, with a focus on MLTC being as important as kidney protection.

## Supplementary Material

Supplementary file .docx

## Data Availability

Due to a data sharing agreement between the University of Nottingham and University of Southampton, these data are unable to be shared directly. However, investigators may apply to the corresponding author to request data and access the data from NURTuRE *via* the website at https://nurturebiobank.org.
