# Supplementary material for: The impact of multiple long-term conditions on mortality, progression to kidney failure and health-related quality of life among people with chronic kidney disease: a multicentre cohort study (NURTuRE-CKD)
Source: Ann Med. 2026 Jun 23;58(1):2692812. doi: 10.1080/07853890.2026.2692812 (PMC13292312; doi:10.1080/07853890.2026.2692812)
Supplement: Supplementary file .docx [file IANN_A_2692812_SM9857.docx]

**Supplementary table 1: Number of people at risk at selected time intervals**

| **Time interval**  **(days)** | **Number of comorbidities** | | | | | **Total** |
| --- | --- | --- | --- | --- | --- | --- |
|  | **One** | **Two** | **Three** | **Four** | **Five +** |  |
| 0 | 514 | 650 | 619 | 431 | 782 | 2996 |
| 250 | 509 | 642 | 614 | 426 | 754 | 2945 |
| 500 | 505 | 630 | 597 | 410 | 720 | 2862 |
| 750 | 499 | 620 | 569 | 399 | 681 | 2768 |
| 1000 | 494 | 608 | 551 | 374 | 635 | 2662 |
| 1250 | 481 | 582 | 518 | 348 | 590 | 2519 |
| 1500 | 328 | 410 | 375 | 251 | 427 | 1791 |
| 1750 | 123 | 151 | 136 | 81 | 127 | 618 |
| 2000 | 13 | 16 | 14 | 8 | 5 | 56 |

**Supplementary table 2: Association between number of baseline comorbidities and CKD progression**

|  |  | **Unadjusted** | | | **Multivariable** | | |
| --- | --- | --- | --- | --- | --- | --- | --- |
|  |  | **HR** | **CI** | **P value** | **HR** | **CI** | **P value** |
| Comorbidities | 1(reference) | | | | | | |
|  | 2 | 1.27 | (1.00 to 1.61) | 0.052 | 1.14 | (0.88 to 1.47) | 0.324 |
|  | 3 | 1.10 | (0.86 to 1.42) | 0.436 | 0.84 | (0.63 to 1.11) | 0.213 |
|  | 4 | 0.96 | (0.72 to 1.27) | 0.777 | 0.79 | (0.57 to 1.08) | 0.140 |
|  | ≥5 | 1.14 | (0.90 to 1.45) | 0.285 | 0.91 | (0.67 to 1.23) | 0.523 |
| Age |  | 1.00 | (0.99 to 1.00) | 0.457 | 0.97 | (0.97 to 0.98) | <0.001 |
| Sex (vs Male) | Female | 0.69 | (0.59 to 0.81) | < 0.001 | 0.78 | (0.65 to 0.93) | 0.005 |
| Ethnicity (vs white) | Asian | 1.21 | (0.92 to 1.60) | 0.177 | 1.39 | (1.02 to 1.88) | 0.034 |
|  | Black | 1.45 | (0.99 to 2.14) | 0.213 | 1.50 | (0.96 to 2.33) | 0.072 |
|  | Mixed | 0.87 | (0.41 to 1.82) | 0.703 | 0.79 | (0.35 to 1.79) | 0.572 |
|  | Other | 0.45 | (0.20 to 1.01) | 0.052 | 0.20 | (0.05 to 0.80) | 0.023 |
| Socioeconomic  Deprivation (by quintile of Index of Multiple Deprivation (IMD) | 1 most deprived (reference) | | | | | | |
|  | 2 | 1.04 | (0.83 to 1.32) | 0.726 | 1.00 | (0.79 to 1.30) | 0.942 |
|  | 3 | 1.06 | (0.84 to 1.35) | 0.620 | 0.91 | (0.70 to 1.18) | 0.467 |
|  | 4 | 1.02 | (0.80 to 1.30) | 0.902 | 0.82 | (0.63 to 1.08) | 0.161 |
|  | 5 | 1.16 | (0.92 to 1.46) | 0.210 | 1.02 | (0.78 to 1.33) | 0.883 |
| Education (Vs None) | GCSE/NVQ/A level^b^ | 0.90 | (0.76 to 1.07) | 0.590 | 0.93 | (0.76 to 1.14) | 0.494 |
|  | Higher education | 0.75 | (0.61 to 0.92) | 0.006 | 0.91 | (0.72 to 1.17) | 0.471 |
| Smoking | Ex smoker | 1.07 | (0.91 to 1.26) | 0.405 | 1.13 | (0.95 to 1.35) | 0.163 |
|  | Current smoker | 1.14 | (0.87 to 1.49) | 0.355 | 1.40 | (1.04 to 1.88) | 0.026 |
| Alcohol use | Yes | 0.92 | (0.79 to 1.07) | 0.278 | 1.03 | (0.86 to 1.23) | 0.743 |
| Number of medicines |  | 1.06 | (1.04 to 1.07) | < 0.001 | 1.03 | (1.00 to 1.05) | 0.017 |
| Mapped 3L index |  | 0.71 | (0.53 to 0.95) | 0.021 | 1.02 | (0.70 to 1.48) | 0.929 |
| eGFR mL/min/1.73 m2 |  | 0.88 | (0.87 to 0.89) | < 0.001 | 0.88 | (0.86 to 0.88) | <0.001 |
| uACR mg/g |  | 1.00 | (1.00 to 1.00) | <0.001 | 1.00 | (1.00 to 1.00) | 0.006 |

a Model adjusted for age, sex, ethnicity, socioeconomic status, alcohol use, educational status, smoking status, number of medicines, mapped EQ-5D-3L index, eGFR, uACR. Region of recruitment was added as a random effect (shared frailty).

b General Certificate of Secondary Education, National Vocational Qualification, Advance level certificate

**Supplementary table 3: Association between the number of baseline comorbidities and health-related quality of life at baseline**

|  |  | **Unadjusted** | | | **Multivariable^a^** | | |
| --- | --- | --- | --- | --- | --- | --- | --- |
|  |  | **Coefficient** | **95% Confidence interval** | **P value** | **Coefficient** | **95% Confidence interval** | **P value** |
| **Number of comorbidities (vs. 1)** | 2 | -0.04 | (-0.07 to -0.01) | 0.004 | -0.02 | (-0.05 to 0.01) | 0.159 |
|  | 3 | -0.08 | (-0.11 to -0.05) | < 0.001 | -0.03 | (-0.06 to -0.00) | 0.027 |
|  | 4 | -0.13 | (-0.16 to -0.10) | < 0.001 | -0.07 | (-0.10 to -0.04) | < 0.001 |
|  | ≥5 | -0.26 | (-0.28 to -0.23) | < 0.001 | -0.14 | (-0.17 to -0.11) | < 0.001 |
| **Age** |  | -0.00 | (-0.00 to -0.00) | < 0.001 | -0.00 | (-0.00 to 0.00) | 0.318 |
| **Sex (vs. Male)** | Female | -0.06 | (-0.08 to -0.04) | < 0.001 | -0.05 | (-0.07 to -0.04) | < 0.001 |
| **Ethnicity (vs. White)** | Asian | 0.00 | (-0.04 to 0.04) | 0.982 | -0.02 | (-0.05 to 0.01) | 0.261 |
|  | Black | -0.02 | (-0.07 to 0.04) | 0.489 | -0.02 | (-0.07 to 0.03) | 0.350 |
|  | Mixed | 0.02 | (-0.07 to 0.11) | 0.610 | 0.01 | (-0.08 to 0.09) | 0.900 |
|  | Other | 0.09 | (0.02 to 0.16) | 0.012 | 0.07 | (0.01 to 0.14) | 0.019 |
| **Socioeconomic status (by quintile of Index of Multiple Deprivation (IMD**) | 1 most deprived (reference) | | | | | | |
|  | 2 | -0.01 | (-0.03 to 0.02) | 0.634 | 0.01 | (-0.02 to 0.03) | 0.580 |
|  | 3 | -0.05 | (-0.08 to -0.02) | 0.001 | -0.02 | (-0.05 to 0.00) | 0.099 |
|  | 4 | -0.06 | (-0.09 to -0.03) | < 0.001 | -0.03 | (-0.05 to -0.00) | 0.048 |
|  | 5 | -0.12 | (-0.15 to -0.10) | < 0.001 | -0.07 | (-0.09 to 0.04) | < 0.001 |
| **Educational attainment (vs. none)** | GCSE/NVQ/A level^b^ | 0.08 | (0.05 to 0.10) | < 0.001 | 0.04 | (0.02 to 0.06) | 0.001 |
|  | Higher education | 0.14 | (0.12 to 0.17) | < 0.001 | 0.06 | (0.04 to 0.09) | < 0.001 |
| **Smoking (vs None)** | Ex smoker | -0.05 | (-0.07 to -0.03) | < 0.001 | -0.03 | (-0.05 to -0.01) | 0.001 |
|  | Current smoker | -0.07 | (-0.10 to -0.03) | < 0.001 | -0.04 | (-0.08 to -0.01) | 0.005 |
| **Alcohol use (vs No** | Yes | 0.10 | (0.08 to 0.12) | < 0.001 | 0.05 | (0.03 to 0.06) | < 0.001 |
| **Number of medications** |  | -0.02 | (-0.02 to - 0.02) | < 0.001 | -0.01 | (-0.02 to -0.01) | < 0.001 |
| **eGFR mL/min/1.73 m2** |  | 0.001 | (0.001 to 0.002) | < 0.001 | 0.00 | (-0.00 to 0.00) | 0.403 |
| **uACR mg/g** |  | -0.00 | (-0.00 to 0.00) | 0.356 | -0.00 | (-0.00 to 0.00) | 0.983 |

a Model adjusted for age, sex, ethnicity, socioeconomic status, alcohol use, educational status, smoking status, number of medicines, eGFR, uACR. Region of recruitment was added as a random effect (random intercepts).

b General Certificate of Secondary Education, National Vocational Qualification, Advance level certificate

**Supplementary table 4: Association between the number of baseline comorbidities and health-related quality of life at second follow up**

|  |  | **Unadjusted** | | | **Multivariable^a^** | | |
| --- | --- | --- | --- | --- | --- | --- | --- |
|  |  | **Coefficient** | **95% Confidence interval** | **P value** | **Coefficient** | **95% Confidence interval** | **P value** |
| **Number of comorbidities (vs. 1)** | 2 | -0.03 | (-0.08 to 0.02) | 0.255 | -0.01 | (-0.06 to 0.04) | 0.648 |
|  | 3 | -0.08 | (-0.13 to -0.03) | 0.003 | -0.05 | (-0.10 to -0.00) | 0.034 |
|  | 4 | -0.08 | (-0.14 to -0.02) | 0.005 | -0.03 | (-0.08 to 0.03) | 0.386 |
|  | ≥5 | -0.21 | (-0.26 to -0.16) | < 0.001 | -0.09 | (-0.15 to -0.04) | 0.001 |
| **Age** |  | -0.003 | (-0.004 to -0.001) | < 0.001 | -0.001 | (-0.003 to 0.00) | 0.101 |
| **Sex (vs. Male)** | Female | -0.05 | (-0.09 to -0.02) | 0.002 | -0.05 | (-0.08 to -0.02) | 0.003 |
| **Ethnicity (vs. White)** | Asian | -0.04 | (-0.12 to 0.05) | 0.406 | -0.02 | (-0.10 to 0.06) | 0.581 |
|  | Black | -0.02 | (-0.14 to 0.09) | 0.699 | 0.004 | (-0.10 to 0.11) | 0.949 |
|  | Mixed | 0.05 | (-0.14 to 0.24) | 0.582 | -0.05 | (-0.25 to 0.15) | 0.617 |
|  | Other | 0.02 | (-0.15 to 0.19) | 0.829 | 0.06 | (-0.10 to 0.21) | 0.468 |
| **Socioeconomic status (by quintile of Index of Multiple Deprivation (IMD**) | 1 most deprived (reference) | | | | | | |
|  | 2 | -0.005 | (-0.05 to 0.04) | 0.838 | 0.02 | (-0.02 to 0.06) | 0.384 |
|  | 3 | -0.03 | (-0.08 to 0.02) | 0.190 | 0.006 | (-0.04 to 0.05) | 0.821 |
|  | 4 | -0.07 | (-0.12 to -0.01) | 0.014 | -0.02 | (-0.07 to 0.03) | 0.344 |
|  | 5 | -0.15 | (-0.20 to -0.10) | < 0.001 | -0.07 | (-0.12 to -0.02) | 0.012 |
| **Educational attainment (vs. none)** | GCSE/NVQ/A level^b^ | 0.11 | (0.07 to 0.16) | < 0.001 | 0.08 | (0.03 to 0.12) | < 0.001 |
|  | Higher education | 0.19 | (0.15 to 0.24) | < 0.001 | 0.11 | (0.06 to 0.15) | < 0.001 |
| **Smoking (vs None)** | Ex smoker | -0.05 | (-0.09 to -0.02) | 0.002 | -0.04 | (-0.07 to -0.003) | 0.032 |
|  | Current smoker | -0.20 | (-0.27 to - 0.12) | < 0.001 | -0.17 | (-0.24 to -0.10) | < 0.001 |
| **Alcohol use (vs No** | Yes | 0.11 | (0.08 to 0.14) | < 0.001 | 0.06 | (0.02 to 0.09) | 0.001 |
| **Number of medications** |  | -0.02 | (-0.02 to -0.02) | < 0.001 | -0.01 | (-0.02 to -0.01) | < 0.001 |
| **eGFR mL/min/1.73 m2** |  | 0.001 | (0.000 to 0.002) | 0.042 | 0.00 | (-0.00 to 0.00) | 0.781 |
| **uACR mg/g** |  | -0.00 | (-0.00 to 0.00) | 0.473 | 0.00 | (-0.00 to 0.00) | 0.317 |

a Model adjusted for age, sex, ethnicity, socioeconomic status, alcohol use, educational status, smoking status, number of medicines, eGFR, uACR. Region of recruitment was added as a random effect (random intercepts).

b General Certificate of Secondary Education, National Vocational Qualification, Advance level certificate.

**Supplementary table 5: Association between specific baseline conditions and health-related quality of life at second follow up**

|  |  | **Unadjusted** | | | **Multivariable^a^** | | | |
| --- | --- | --- | --- | --- | --- | --- | --- | --- |
|  |  | **Coefficient** | **95% Confidence Interval** | **P value** | **Coefficient** | **95% Confidence Interval** | **P value** | |
| **Comorbidities** | PVD | -0.25 | (-0.36 to -0.16) | < 0.001 | -0.14 | (-0.22 to -0.06) | < 0.001 | |
|  | Cerebrovascular  disease | -0.11 | (-0.18 to -0.03) | < 0.001 | -0.02 | (-0.08 to 0.04 | 0.53 | |
|  | Respiratory | -0.08 | (-0.13 to -0.03 | < 0.001 | -0.02 | (-0.06 to 0.02) | 0.39 | |
|  | Gastrointestinal | -0.11 | (-0.21 to -0.00) | 0.04 | 0.00 | (-0.08 to 0.08) | 0.91 | |
|  | Diabetes and complications | -0.13 | (-0.17 to -0.08) | < 0.001 | -0.02 | (-0.06 to 0.02) | 0.41 | |
|  | Hypertension | -0.02 | (-0.07 to 0.03) | 0.41 | -0.01 | (-0.03 to 0.05) | 0.54 | |
|  | Heart failure | -0.09 | (-0.18 to 0.00) | 0.05 | 0.03 | (-0.05 to 0.11) | 0.54 | |
|  | AF | -0.09 | (-0.15 to -0.02) | 0.01 | -0.02 | (-0.07 to 0.04) | 0.51 | |
|  | Obesity | **-**0.11 | (-0.14 to -0.07) | < 0.001 | -0.06 | (-0.09 to -0.02) | < 0.001 | |
|  | Pain | -0.19 | (-0.22 to -0.15) | < 0.001 | -0.11 | (-0.14 to -0.08) | < 0.001 | |
|  | Sarcopenia | -0.16 | (-0.20 to -0.11) | < 0.001 | -0.09 | (-0.13 to -0.05) | < 0.001 | |
|  | Connective tissue disease + Autoimmune disease | -0.04 | (-0.09 to 0.02) | 0.23 | -0.00 | (-0.04 to 0.03) | 0.86 | |
|  | Anemia | -0.07 | (-0.18 to 0.04) | 0.19 | 0.11 | (-0.08 to 0.09) | 0.80 | |
|  | Cancer | -0.02 | (-0.07 to 0.03) | 0.43 | -0.02 | (-0.06 to 0.02) | 0.33 | |
|  | Liver disease | -0.04 | (-0.11 to 0.04) | 0.31 | 0.01 | (-0.06 to 0.07) | 0.80 | |
|  | Hyperuricemia | -0.04 | (-0.08 to 0.00) | 0.08 | -0.03 | (-0.06 to 0.01) | 0.11 | |
|  | Mental health | -0.21 | (-0.25 to - 0.17) | < 0.001 | -0.13 | (-0.16 to -0.09) | < 0.001 | |
|  | Cognitive impairment | -0.08 | (-016 to 0.00) | 0.06 | -0.02 | (-0.08 to 0.04) | 0.51 | |
|  | IHD | -0.13 | (0.01 to 0.07) | < 0.001 | -0.04 | (-0.10 to 0.02) | 0.16 | |
| **Age** |  | -0.003 | (-0.00 to -0.001) | < 0.001 | -0.00 | (-0.00 to 0.00) | 0.05 | |
| **Sex** |  | -0.05 | (-0.08 to -0.02) | < 0.001 | -0.04 | (-0.07 to -0.01) | 0.02 | |
| **Ethnicity** | Asian | -0.00 | (-0.09 to 0.08) | 0.92 | 0.03 | (-0.06 to 0.11) | 0.52 | |
|  | Black | 0.01 | (-0.12 to 0.13) | 0.93 | 0.08 | (-0.04 to 0.19) | 0.17 | |
|  | Mixed | -0.01 | (-0.25 to 0.23) | 0.93 | -0.07 | (-0.29 to 0.14) | 0.56 | |
|  | Other | -0.00 | (-0.19 to 0.19) | 0.98 | 0.12 | (-0.04 to 0.27) | 0.14 | |
| **Socioeconomic**  **Deprivation (by quintile of Index of Multiple Deprivation (IMD)** | 1 most deprived (reference) | | | | | | |  |
|  | 2 | 0.08 | (0.02 to 0.14) | < 0.001 | 0.03 | (-0.02 to 0.08) | 0.20 | |
|  | 3 | 0.11 | (0.05 to 0.16) | < 0.001 | 0.05 | (-0.01 to 0.09) | 0.09 | |
|  | 4 | 0.14 | (0.09 to 0.19) | < 0.001 | 0.06 | (0.02 to 0.12) | 0.01 | |
|  | 5 | 0.14 | (0.09 to 0.19) | < 0.001 | 0.03 | (-0.02 to 0.08) | 0.20 | |
| **Educational attainment (vs none)** | GCSE/NVQ/A level^b^ | 0.12 | (0.07 to 0.16) | < 0.001 | 0.05 | (0.01 to 0.09) | 0.01 | |
|  | Higher education | 0.20 | (0.15 to 0.24) | < 0.001 | 0.07 | (0.02 to 0.11) | < 0.001 | |
| **Smoking** | Ex smoker | -0.05 | (-0.09 to -0.02) | < 0.001 | -0.03 | (-0.06 to 0.00) | 0.08 | |
|  | Current smoker | -0.20 | (-0.28 to -0.13) | < 0.001 | 0.13 | (-0.02 to -0.06) | < 0.001 | |
| **Alcohol use (vs None)** | Yes | 0.11 | (0.07 to 0.14) | < 0.001 | 0.04 | (-0.01 to 0.07) | < 0.001 | |
| **Number of medications** |  | -0.02 | (-0.02 to -0.02) | < 0.001 | -0.01 | (-0.01 to -0.00) | < 0.001 | |
| **eGFR mL/min/1.73 m2** |  | 0.001 | (0.00 to 0.002) | 0.04 | 0.00 | (-0.00 to 0.00) | 0.82 | |
| **uACR mg/g** |  | -0.00 | (-0.00 to 0.00) | 0.50 | 0.00 | (-0.00 to 0.00) | 0.29 | |

a Model adjusted for age, sex, ethnicity, socioeconomic status, alcohol use, educational status, smoking status, number of medicines, eGFR, uACR. Region of recruitment was added as a random effect (random intercepts).

b General Certificate of Secondary Education, National Vocational Qualification, Advance level certificate.

**Supplementary Table 6: Baseline characteristics of NURTuRE-CKD participants split by whether they returned for the first follow-up or not.**

Unless otherwise stated, all variables are presented as number and percentage

|  |  | **Returned for first follow-up**  **(N=2062)** | **No first follow-up**  **(N=934)** | **P value** |
| --- | --- | --- | --- | --- |
| **Age** | Mean (SD)  Median  LQ to UQ  Min to Max | 61.92 (14.43)  65  53 to 73  18 to 94 | 64.15 (15.32)  68  54 to 76  18 to 95 | **<0.001^1^** |
| **Sex** | Female | 866 (42.0%) | 377 (40.36%) | 0.400^2^ |
|  | Male | 1196 (58.0%) | 557 (59.6%) |  |
| **Ethnicity** | Asian | 136 (6.6%) | 64 (6.9%) | 0.080^2^ |
|  | Black | 53 (2.6%) | 38 (4.1%) |  |
|  | Mixed | 22 (1.1%) | 12 (1.3%) |  |
|  | Other | 31 (1.5%) | 22 (2.4%) |  |
|  | White | 1817 (88.3%) | 796 (85.4%) |  |
| **Socioeconomic status (by quintile of Index of Multiple Deprivation (IMD)** | 1 (most deprived) | 483 (23.5%) | 163 (17.5%) | **0.001^2^** |
|  | 2 | 433 (21.1%) | 184 (19.7%) | **<0.001^1^** |
|  | 3 | 377 (18.3%) | 180 (19.3%) |  |
|  | 4 | 350 (17.0%) | 200 (21.4%) |  |
|  | 5 (least deprived) | 414 (20.1%) | 206 (22.1%) |  |
| **Educational attainment** | None | 512 (24.8%) | 350 (37.5%) | **<0.001^2^** |
|  | GCSE/NVQ/A levels^*^ | 977 (47.4%) | 374 (40.0%) | **<0.001^1^** |
|  | Higher education | 573 (27.8%) | 210 (22.5%) |  |
| **Smoking status** | Nonsmoker | 1070 (57.3%) | 413 (45.5%) | **<0.001^2^** |
|  | Ex smoker | 813 (39.7%) | 396 (43.6%) |  |
|  | Current smoker | 164 (8.0%) | 99 (10.9%) |  |
| **Alcohol intake** | None | 896 (44.1%) | 474 (52.4%) | **<0.001^2^** |
|  | Any | 1137 (55.9%) | 430 (47.6%) |  |
| **Number of medications** | Mean (SD)  Median  LQ to UQ  Min to Max | 8.17 (4,72)  7  5 to 11  1 to 33 | 6.93  7  4 to 9  1 to 32 | **<0.001^1^** |
| **eGFR mL/min/1.73 m2** | Mean (SD)  Median  LQ to UQ  Min to Max | 38.46 (17.84)  34.95  25.36 to 47.97  6.18 to 127.56 | 34.69 (17.64)  31.26  21.60 to 43.47  8.36 to 133.97 | **<0.001^1^** |
| **Urine albumin creatinine ratio (uACR) mg/g** | Mean (SD)  Median  LQ to UQ  Min to Max | 85.32 (283.49)  20.62  3.57 to 97.89  0.12 to 10990.61 | 101.34 (171.88)  29.59  3.96 to 117.83  0.27 to 1501.17 | **0.009^1^** |
| **Number of comorbidities** | Mean (SD)  Median  LQ to UQ  Min to Max | 3.53 (2.19)  3  2 to 5  1 to 19 | 3.39 (2.14)  3  2 to 4  1 to 14 | 0.093^1^ |
|  | 1 | 340 (16.5%) | 174 (18.6%) | 0.150^2^ |
|  | 2+ | 1722 (83.5%) | 760 (81.4%) |  |

*General Certificate of Secondary Education, National Vocational Qualification, Advance level certificate

^1^Mann-Whitney U test.

^2^Chi-square test.

Statistically significant results are displayed in bold.

**Supplementary Table 7: Baseline characteristics of NURTuRE-CKD participants split by whether they returned the Questionnaire follow-up or not.**

Unless otherwise stated, all variables are presented as number and percentage

|  |  | **Returned Questionnaire follow-up**  **(N=1019)** | **No Questionnaire follow-up**  **(N=1977)** | **P value** |
| --- | --- | --- | --- | --- |
| **Age** | Mean (SD)  Median  LQ to UQ  Min to Max | 63.59 (12.29)  66  56 to 72  19 to 89 | 62.11 (15.85)  65  51 to 75  18 to 95 | 0.384^1^ |
| **Sex** | Female | 444 (43.6%) | 799 (40.4%) | 0.097^2^ |
|  | Male | 575 (56.4%) | 1178 (59.6%) |  |
| **Ethnicity** | Asian | 41 (4.0%) | 159 (8.1%) | **<0.001**^2^ |
|  | Black | 23 (2.3%) | 68 (3.4%) |  |
|  | Mixed | 8 (0.8%) | 26 (1.3%) |  |
|  | Other | 10 (1.0%) | 43 (2.2%) |  |
|  | White | 935 (91.9%) | 1678 (85.0%) |  |
| **Socioeconomic status (by quintile of Index of Multiple Deprivation (IMD)** | 1 (most deprived) | 274 (26.9%) | 372 (18.9%) | **<0.001**^2^ |
|  | 2 | 241 (23.7%) | 376 (19.1%) | **<0.001**^1^ |
|  | 3 | 183 (18.0%) | 374 (19.0%) |  |
|  | 4 | 168 (16.5%) | 382 (19.4%) |  |
|  | 5 (least deprived) | 152 (14.9%) | 468 (23.7%) |  |
| **Educational attainment** | None | 227 (22.3%) | 635 (32.1%) | **<0.001**^2^ |
|  | GCSE/NVQ/A levels^*^ | 459 (45.0%) | 892 (45.1%) | **<0.001**^1^ |
|  | Higher education | 333 (32.7%) | 450 (22.8%) |  |
| **Smoking status** | Nonsmoker | 573 (56.6%) | 910 (46.8%) | **<0.001**^2^ |
|  | Ex smoker | 385 (38.0%) | 824 (42.4%) |  |
|  | Current smoker | 54 (5.3%) | 209 (10.8%) |  |
| **Alcohol intake** | None | 400 (39.6%) | 970 (50.3%) | **<0.001**^2^ |
|  | Any | 609 (60.4%) | 958 (49.7%) |  |
| **Number of medications** | Mean (SD)  Median  LQ to UQ  Min to Max | 7.17 (4.08)  7  4 to 10  1 to 28 | 8.10 (4.68)  7  5 to 11  1 to 33 | **<0.001**^1^ |
| **eGFR mL/min/1.73 m2** | Mean (SD)  Median  LQ to UQ  Min to Max | 38.67 (16.32)  35.96  26.69 to 47.43  11.63 to 114.13 | 36.57 (18.57)  32.63  22.79 to 45.28  6.18 to 133.97 | **<0.001**^1^ |
| **Urine albumin creatinine ratio (uACR) mg/g** | Mean (SD)  Median  LQ to UQ  Min to Max | 67.12 (122.28)  14.03  2.69 to 70.48  0.21 to 882.50 | 102.50 (301.77)  29,82  4.87 to 116.84  0.12 to 10990.61 | **<0.001**^1^ |
| **Number of comorbidities** | Mean (SD)  Median  LQ to UQ  Min to Max | 3.25 (2.02)  3  2 to 4  1 to 15 | 3.61 (2.25)  3  2 to 5  1 to 19 | **<0.001**^1^ |
|  | 1 | 195 (19.1%) | 319 (16.1%) | **0.039**^2^ |
|  | 2+ | 824 (80.9%) | 1658 (83.9%) |  |

*General Certificate of Secondary Education, National Vocational Qualification, Advance level certificate

^1^Mann-Whitney U test.

^2^Chi-square test.

Statistically significant results are displayed in bold.

**Supplementary Figure 1: Cause specific Cumulative Incidence plot of progression by number of comorbidities**

**Supplementary Figure 2: Unadjusted association between number of comorbidities and health-related quality of life (using EQ-5D-3L index values) at first follow up**

**
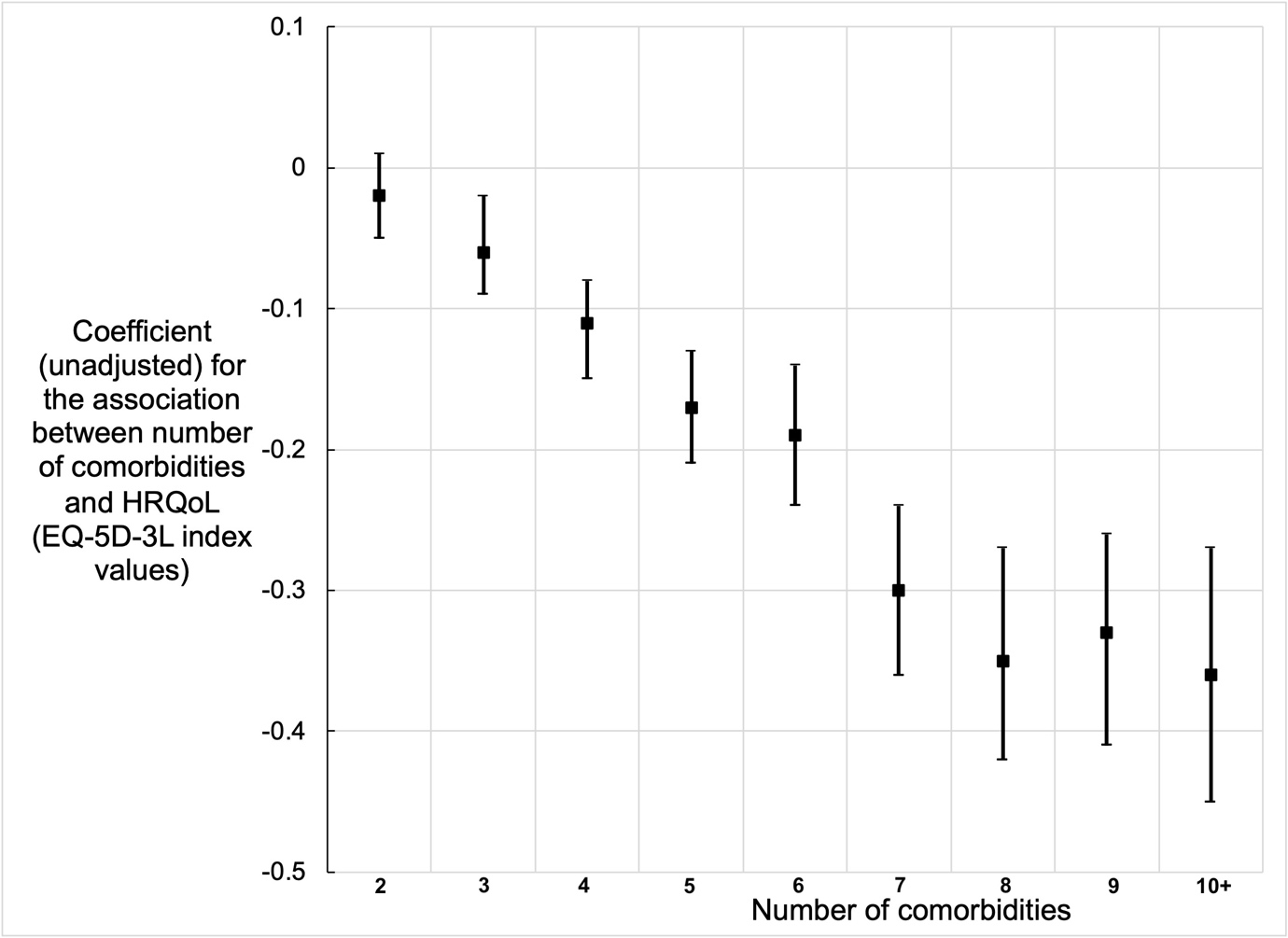
**
